# Respiratory syncytial virus infection: a new threat of public health, epidemiology, pathogenesis, genomic characteristics, and current status

**DOI:** 10.1097/JS9.0000000000000197

**Published:** 2023-01-23

**Authors:** Shopnil Akash, Talha B. Emran, Ruhul Amin, Kuldeep Dhama

**Affiliations:** a Department of Pharmacy, Faculty of Allied Health Sciences, Daffodil International University, Dhaka; b Department of Pharmacy, BGC Trust University Bangladesh, Chittagong, Bangladesh; c Faculty of Pharmaceutical Science, Assam Down Town University, Panikhaiti, Guwahati, Assam; d Division of Pathology, ICAR-Indian Veterinary Research Institute, Bareilly, Izatnagar, Uttar Pradesh, India

Highlights

- Respiratory syncytial virus (RSV) is associated with a high fatality rate and a large number of morbidities.
- RSV infection affects around two-thirds of newborns during their first year of life.
- RSV is an enshrouded, medium-sized RNA virus.
- RSV cultures are another diagnostic option but have limited relevance due to the time.

*Dear Editor*,

The respiratory syncytial virus (RSV), often known as RSV, is one of the most life-threatening microorganisms that may affect younger children. It is also associated with a high fatality rate and a large number of morbidities^1^. Newborns and young children are mostly infected by this life-threatening infection all around the globe, which is caused by this RSV. Besides, it directly impacts human health and is responsible for several million deaths each year among children under the age of 5 all over the globe^2^. Additionally, it is accountable for spending hundreds of millions of dollars in healthcare systems to treat infected patients. Another study displayed that the lower respiratory tract infections caused by RSV are responsible for an estimated 100,000–125,000 hospitalizations and up to 450 fatalities annually in the United States. It may be detrimental for babies who are born prematurely as well as for those who already have chronic lung illness. In addition, research has found that children who are exposed to infection when they are infants are more likely to suffer from hyperreactive airways and asthma later in life^3^. RSV infection affects around two-thirds of newborns during their first year of life; by the age of 2 years, 90% of children have had at least one RSV infection. The incidence of hospitalization due to primary infection is typically 0.5%. However, this number might change depending on the patient’s circumstances and ethnicity, reaching as high as 25%^4^.

Although in 1956, the RSV virus that causes upper respiratory tract infection in humans was first identified from a chimpanzee in a lab, at the time, it was not initially connected to respiratory illnesses in newborns. When it was seen that a group of 14 chimpanzees was suffering from colds and coryza, Morris and his co-workers identified a novel virus first referred to as the chimpanzee coryza agent (CCA)^5^. The term ‘chimpanzee coryza agent’ replaced RSV after it was discovered that the majority of school-aged children had a particular antibody that could neutralize CCA^6^,^7^. Subsequently, Chanock and co-workers acquired isolates from two children, one with laryngotracheobronchitis and the other with bronchopneumonia, that were indistinguishable from CCA. This served as confirmation that the agent triggered respiratory disease in people.

Before the discovery of coronavirus disease, the RSV was a relatively unknown virus. However, the incidence of this impacted almost every aspect of pneumonia, and bronchiolitis (inflammation of the airways) is rising, leading to a shortage of hospital beds in Europe and the Americas. When coupled with growing hospitalizations for other respiratory diseases such as influenza and coronavirus disease, it brings certain healthcare facilities dangerously near the point of failure. According to the European Centre for Disease Prevention and Control’s most recent news release, a wide range of countries have been beginning to experience extremely early increment in RSV observations, which has caused an increase in the number of pediatric hospital admissions in France, Ireland, Spain, Sweden, and the United States. The United States of America had persistently high rates of RSV infections from July 2021 through February 2022, followed by a further rise during July and August 2022. Now, they are climbing at an alarming pace, with RSV hospitalization rates for babies being seven times greater than in 2018^6^.

The RSV is an enshrouded, medium-sized (120–300 nm) RNA virus. It has a nonsegmental, single-stranded, negative-sense genome affiliated with viral proteins across its length, constituting the nucleocapsid. The bilipid layer of the viral particle is thought to have originated from the plasma membrane of the host cells. The area is coated with transmembrane surface glycoprotein spikes that range in length from 11 to 12 nm and is spaced between 6 and 10 nm, which, when seen via an electron microscope, give it a thistle-like look^7^,^8^.

Respiratory secretions may transmit RSV (coughing, sneezing, or kissing) from a person who is sick or via encountering materials that are exposed to the virus and then contacting one’s eyes, nose, or mouth. RSV can survive on hard surfaces for a significant amount of time, such as tables and crib rails. In most cases, the virus can only stay on soft surfaces like tissues and palms for brief periods. Children are often exposed to RSV in environments other than their homes, such as schools and childcare facilities. They can then pass the infection on to other family members^9^.

The concept of clinical diagnosis is the activity of discovering the nature of a disease or disorder and differentiating it from other probable diseases, and deciding which illness or condition adequately describes the indications and manifestations that a patient is experiencing^10^. So, the results of RSV diagnostic procedures are often informative, but they seldom lead to a change in therapy. Although nasopharyngeal washes or tracheal secretions provide more reliable specimens for confirming RSV1, nasal swabs remain the method of choice owing to their simplicity of collection. Due to its cheap cost, quick turnaround (30 min), and objective endpoint, enzyme immunoassay is the most used fast detection test. When RSV is circulating in a given area, a sensitivity of 50–90% may be expected from an enzyme immunoassay of 13, although it has a specificity of 90–95%. Negative test results should not be used to dismiss the possibility of RSV in the presence of clinical symptoms. Immunofluorescent tests, both direct and indirect, are possible but need a lot of time and specialized staff to complete. RSV cultures are another diagnostic option but have limited relevance due to the time, money, and methodological uncertainty involved^11^.

Although there is currently no cure for RSV infection, scientists are actively working on developing vaccinations and antiviral medications (medicines that fight viruses). Patients with RSV infections have thus been subjected to several palliative therapies; nevertheless, the efficiency of most of these treatments is still not well proven. In addition, the alternative medicine for the RSV is taking care of your child directly to help them feel more at ease (supportive care). However, medical treatment may be necessary if the symptoms are severe^12^,^13^.

## Ethical approval

Not applicable.

## Sources of funding

None.

## Author contribution

S.A.: conceptualization, data curation, writing – original draft preparation, and writing – reviewing and editing; T.B.E.: writing – reviewing and editing, visualization, and supervision; R.A.: data curation, writing – original draft preparation, and writing – reviewing and editing; K.D.: data curation, writing – original draft preparation, and writing – reviewing and editing.

## Conflicts of interest disclosure

The authors declare that they have no conflicts of interest.

## Research registration unique identifying number (UIN)

1. Name of the registry: not applicable.

2. Unique identifying number or registration ID: not applicable.

3. Hyperlink to your specific registration (must be publicly accessible and will be checked): not applicable.

## Guarantor

T.B. Emran, PhD, Associate Professor, Department of Pharmacy, BGC Trust University Bangladesh, Chittagong 4381, Bangladesh. Tel: +880 303 356 193, fax: +880 312 550 224. https://orcid.org/0000-0003-3188-2272.

## Provenance and peer review

Not commissioned, internally peer-reviewed.

## Data availability

The data in this correspondence article is not sensitive in nature and is accessible in the public domain. The data is, therefore, available and not of a confidential nature. No specific data were collected for the above manuscript.
